# A Text Message–Based Intervention for Weight Loss: Randomized Controlled Trial

**DOI:** 10.2196/jmir.1100

**Published:** 2009-01-13

**Authors:** Kevin Patrick, Fred Raab, Marc A Adams, Lindsay Dillon, Marian Zabinski, Cheryl L Rock, William G Griswold, Gregory J Norman

**Affiliations:** ^3^Department of Computer Sciences and EngineeringUniversity of CaliforniaSan DiegoLa JollaCAUSA; ^2^Graduate School of Public HealthSan Diego State UniversitySan DiegoCAUSA; ^1^Department of Family and Preventive Medicine and the California Institute of Telecommunications and Information Technology (Calit2)University of CaliforniaSan DiegoLa JollaCAUSA

**Keywords:** Mobile phone, obesity, SMS, text message, health behavior

## Abstract

**Background:**

To our knowledge, no studies have evaluated whether weight loss can be promoted in overweight adults through the use of an intervention that is largely based on daily SMS (Short Message Service: text) and MMS (Multimedia Message Service: small picture) messages transmitted via mobile phones.

**Objective:**

This paper describes the development and evaluation of a text message–based intervention designed to help individuals lose or maintain weight over 4 months.

**Methods:**

The study was a randomized controlled trial, with participants being exposed to one of the following two conditions, lasting 16 weeks: (1) receipt of monthly printed materials about weight control; (2) an intervention that included personalized SMS and MMS messages sent two to five times daily, printed materials, and brief monthly phone calls from a health counselor. The primary outcome was weight at the end of the intervention. A mixed-model repeated-measures analysis compared the effect of the intervention group to the comparison group on weight status over the 4-month intervention period. Analysis of covariance (ANCOVA) models examined weight change between baseline and 4 months after adjusting for baseline weight, sex, and age.

**Results:**

A total of 75 overweight men and women were randomized into one of the two groups, and 65 signed the consent form, completed the baseline questionnaire, and were included in the analysis. At the end of 4 months, the intervention group (n = 33) lost more weight than the comparison group (−1.97 kg difference, 95% CI −0.34 to −3.60 kg, *P* = .02) after adjusting for sex and age. Intervention participants’ adjusted average weight loss was 2.88 kg (3.16%). At the end of the study, 22 of 24 (92%) intervention participants stated that they would recommend the intervention for weight control to friends and family.

**Conclusions:**

Text messages might prove to be a productive channel of communication to promote behaviors that support weight loss in overweight adults.

**Trial Registration:**

Clinicaltrials.gov NCT00415870; http://clinicaltrials.gov/ct2/show/NCT00415870 (Archived by WebCite at http://www.webcitation.org/5dnolbkFt)

## Introduction

The percentage of overweight (body mass index, or BMI, 25.0-29.9 kg/m^2^) and obese (BMI ≥ 30 kg/m^2^) adults has increased in the United States, from 43.3% in 1960 to 66.3% in 2003-04 [1]. Obesity increases the risk of cardiovascular disease [2] and overall mortality [3]. Weight loss as modest as 5% to 10% significantly improves the risk for several chronic diseases, such as hypertension [4], diabetes mellitus and insulin resistance [5], and selected cancers [6]. It has been known for over 30 years that a 10% reduction in weight corresponds to an approximate 20% reduction in coronary disease incidence, whereas a 10% increase in weight is associated with a 30% increase in incidence [7]. Thus, rather than requiring dramatic levels of weight loss, expert consensus is that lifestyle interventions that promote modest levels of sustained weight loss are likely to confer substantial health benefits [8].

Clinical and commercial weight loss programs can produce short-term weight loss, but a majority of adults regain about 40% of the lost weight in the first year and continue to regain [9]. Simple dietary restriction has not been associated with successful weight control [10]. Examining effective approaches to weight loss may be helpful for determining what behavioral skills should be promoted for weight loss and weight loss maintenance. Among these, self-monitoring has emerged as a critical skill for obesity management since those who report monitoring their weight on a daily or weekly basis have greater success in achieving weight loss goals [11,12]. Self-monitoring increases awareness of food and caloric intake, enhances self-efficacy, and allows for monitoring of progress and change over time [13]. However, detailed self-monitoring is labor intensive for participants and adherence is relatively low. One weight loss study found that while self-monitoring of food intake was rated as a useful strategy for weight loss, only 30% of participants continued this behavior after the study had ended [14]. Barriers such as stress, lack of social support, and discomfort with recording can affect adherence to self-monitoring [13].

Mobile phones may provide an opportunity to improve behaviors like self-monitoring, in particular through the use of Short Message Service (SMS), or text, messages. For example, sending text messages to mobile phones increased the effectiveness of a smoking cessation intervention among college students [15]. Another study among young adults in New Zealand revealed that participants who received text messages were more likely to quit smoking at 6 weeks compared to controls [16]. In a program conducted among youth with type 1 diabetes [17], daily text messages were helpful for disease self-management, increased self-efficacy, and treatment adherence and achieved high satisfaction among participants. In a randomized controlled trial of an Internet and mobile phone–based physical activity intervention among overweight adults that included reminders for exercise sessions sent via cell phone, experimental participants engaged in over 2 hours more physical activity per week than those with no access [18]. And, in an uncontrolled study among adults in South Korea, weekly text messages about diet, exercise, and behavior modification proved effective in promoting weight loss at 12 weeks [19]. To date and to our knowledge, there have been no studies on the use of daily text messages delivered via mobile phone as an intervention to address overweight among adults. Thus, we sought to determine if text messaging was a useful and effective strategy to help adults self-monitor their weight and improve weight outcomes. We hypothesized that, compared to overweight adults receiving standard weight control advice, those enrolled in a 4-month program that utilized daily text messages as a means of behavioral prompting, support, and self-monitoring would be more successful in their weight loss goals.

## Methods

### Formative Research

The study evaluated a mobile phone–based application designed as an assessment and intervention tool to improve dietary behaviors and reduce weight. Designing the system began with formative research with overweight men and women to solicit feedback about dietary behaviors, current mobile phone and text and picture message habits, the type and frequency of text and picture messages helpful for weight loss, and nutrition-related topic areas that should be included in a weight loss program. Focus group participants also tested prototypes of the system by receiving and responding to sample text and picture messages. While men and women differed on the preferred number of messages per day, both groups agreed that messages related to motivation, progress with weight loss, tips, and hints would be welcomed.

### Intervention

The system was developed to be both personally tailored and interactive. Personal tailoring was accomplished by providing flexibility in the number and timing of receipt of messages each day. For example, users could choose different times during the day to receive a message—typically one in the morning and one in the evening, with one to three additional messages when the user thinks a reminder would be helpful. A database was developed of over 3000 SMS text and Multimedia Message Service (MMS; essentially small pictures) messages and 1500 rules that determined what message was sent based on the day of week, time of day, and the participant’s eating behaviors and previous replies, among other parameters. Approximately half of the messages requested a reply, with the balance providing tips, suggestions, and positive reinforcement or encouragement for improved behaviors. To minimize annoyance, the system was designed so if the user chose not to respond to a message, it reduced the number of messages requesting replies until a response was provided. Also, the user was given the opportunity to change the time and frequency of the messages after gaining experience with use of the system.

The intervention was organized by topics that changed each week as the participant proceeded through the intervention. The weekly topics included behavioral and dietary strategies known to positively influence weight control, including goal setting and self-monitoring, understanding calories, portion control, pedometers and physical activity, personal strategies for weight loss and overcoming barriers, volumetrics (consuming foods that are healthy and make one feel “full”), replacement and substitution, routine physical activity, organization and meal planning, strategies for eating out, strategies for creating healthy food and exercise environments, strength training, emotion eating, managing tough social situations, body image, and sticking with it. In addition to weekly topics, each week participants were instructed to weigh themselves, fill out worksheets outlining their food and exercise goals, and set goals for a daily 500 calorie reduction using the strategies provided in the corresponding weekly topic. For example, in week 3, participants learned about portion control. Also in week 3, participants were instructed to select portion control strategies that they would use in order to reduce their daily calories. To support self-monitoring, participants used the mobile phone to report their weight once a week. A graph of weight change was also sent to participants weekly.

To keep the text messaging novel and non-repetitive, the type and content of the messages sent throughout the week changed from day to day (Table 1), and participants never got the exact same message throughout the entire duration of the study. As an example, a person requesting two daily messages, one in the morning and the other in the evening, might receive the following:

- a *topic* message on Monday, Wednesday, Thursday, and Saturday, such as “Control your portions by setting aside a large snack package into smaller bags or buy 100 calorie snack packs!”
- a *question* each day asking the participant to reply, such as “How often do you meal plan? A) Every day; D) Every now and then; G) Never.” (A, D, and G were chosen to simplify the use of the key pad for responses since they required only one touch because phones commonly cluster three letters on each button, for example, ABC, DEF, GHI.)
- *tips* or *questions* on Tuesday, Friday, and Sunday that were tailored to the participant’s eating behaviors

For example, a participant who was identified as having difficulty eating fruits and vegetables might get the text message tip “In a rush? Buy pre-cut vegetables like carrots, celery, and mushrooms for a quick, easy, and low calorie snack!” or the question “Did you buy fresh raw vegetables at the grocery store for snacks this week? (y/n)”. If a participant responded to a text message that was a question, he or she would always get a response based on the answer provided. For the above question (“Did you buy fresh raw vegetables at the grocery store for snacks this week?”), if the participant responded “no,” he or she would see the response “Be sure to add your favorite raw veggies like carrots, red peppers, and mushrooms to your shopping list.”

**Table 1:** Representative weekly sequencing of SMS and MMS messages

	Sunday	Monday	Tuesday	Wednesday	Thursday	Friday	Saturday
Morning	Weight Graph	Weekly Topic Tip	Energy Balance (EB) Tip	Weekly Topic Question	Weekly Topic Tip	EB Question	Weekly Topic Tip
Evening	EB Question	Weekly Topic Question	EB Question	Weekly Topic Tip	Weekly Topic Question	EB Tip	Weekly Topic Question

The overall system consists of four components: (1) a Web-based application to enroll participants and set user preferences; (2) a database to store the participants’ records, rules, and messages sent and received; (3) an application to determine the appropriate timing and message to send and to process the received replies; and (4) a text message delivery/reception platform. The system also had tools that enabled continuous technical monitoring to recognize anomalies such as messages and rules missing from the database, logic mistakes, or unexpected responses from participants, indicating that an individual may be having difficulties with the system. These tools alerted the case manager, who could contact the participant by phone or email to prevent user frustration and increase adherence and satisfaction.

A baseline dietary assessment [20] for each participant was used to identify unique diet behavior challenges that may contribute to increased caloric intake (eg, snacking behaviors, pacing of consumption, and self-monitoring of food intake). The server processed these data to create goals to target based upon particular logic rules of the expert system. These goals were then presented to the user via text or MMS messages to serve as prompts for food selection and behavioral improvements. At intervention onset, participants were given a printed binder with nutrition topics and behavioral strategies to supplement the phone-based messaging and a food and exercise journal to support self-monitoring. They also received brief (5-15 minute) monthly phone calls from a trained health counselor to encourage continued participation in the program and to work through any technical issues they might be having with the intervention. During the counseling calls, the health counselor followed a script designed to assess progress and barriers to weight loss. In addition, the health counselor would inquire about any of the following: weekly topics (Which topics are helpful? What have you learned? Is there any information missing?); text messages (Are they helpful?); current physical activity and eating plan (What is your current physical activity plan? How are you cutting calories?); social support (Have you been getting social support from the people around you?); or environment (Have you made any changes to your environment?). The health counselor would then provide feedback on what was discussed during the phone call and offer solutions to overcoming barriers.

### Comparison Group

The usual care comparison group received the same baseline dietary assessment as the intervention group and was mailed one to two pages of print materials once a month for 4 months. The print materials differed from the binder received by the intervention group. Print materials included information and tips on nutrition and weight loss, walking basics, fruit and veggie basics, and how to make physical activity a habit. Some of the topics and content overlapped information provided in intervention group’s binder and text messages. The comparison group did not receive telephone calls or text/MMS messages.

### Study Design

**Figure 1:**
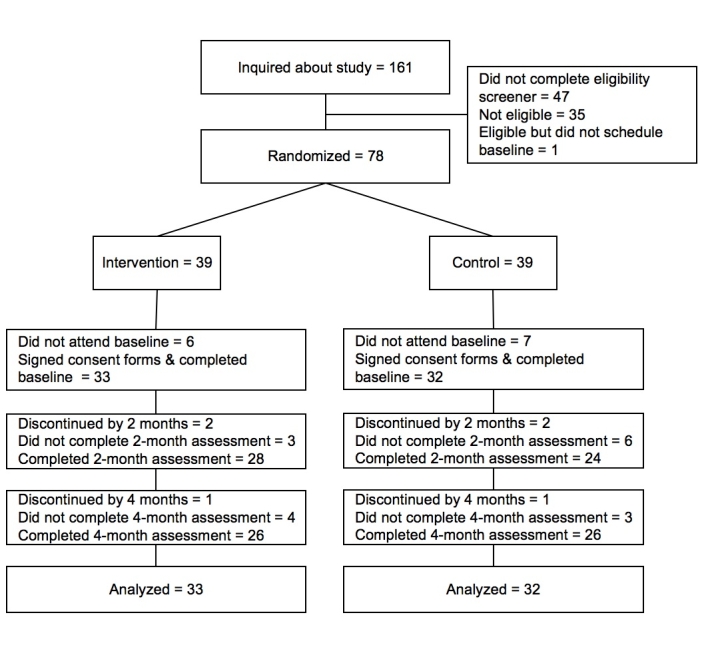
Flow diagram of participants

Evaluation of the system was accomplished in a randomized controlled trial with participants randomized to either the intervention or usual care comparison group (Figure 1). Assessments were completed at baseline, 2 months, and 4 months. The study was approved by the Institutional Review Board at the University of California, San Diego, CA.

### Measures

The primary outcome was weight in kilograms measured in the research offices using a calibrated scale.

### Participants and Recruitment

Participants were recruited in 2007 from the community via newspaper ads, flyers, and announcements on craigslist. Interested individuals called the telephone number provided on the recruitment materials. Recruitment staff answered the telephone and screened potential participants for meeting the following inclusion criteria: 25 to 55 years old, overweight (BMI ≥ 25-39.9), not taking medications known to cause weight gain, and present use of a mobile phone for sending and receiving SMS messages or a willingness to learn. Individuals eligible and interested in the study were transferred to the study staff, who scheduled a baseline visit. During the scheduling process, individuals were assigned to either the comparison or intervention group by a computer-generated process using simple randomization. Participants were not informed of their group allocation status during this process. Following baseline measurements, neither study staff nor participants were blinded to participants’ allocation. As no studies using SMS messages for weight control had been previously published, we did not have guidance on sample size for powering the study, so we based enrollment numbers on our best estimate of likely effects.

### Statistical Analyses

Initial descriptive analyses examined sample characteristics, the randomization of participants, and distribution of variables. A mixed-model repeated-measures analysis compared the effect of the intervention group to the comparison group on weight status over the 4-month intervention period. The mixed-model analysis allowed for inclusion of all available data with missing values assumed missing at random. Model building methods outlined by Singer and Willett [21] were followed. An unconditional means model of baseline weight was evaluated first, followed systematically by models including a within-subject factor of time (0, baseline; 1, 2 months; 2, 4 months), a between-subjects factor of treatment group (1, intervention group; 0, comparison group), and the treatment by time interaction. Further models were planned to include sex, race (white versus non-white), ethnicity (Hispanic versus non-Hispanic), and age (mean centered). Neither race nor ethnicity independently influenced initial weight status or rate of weight change and were dropped from the models. A heterogeneous first-order autoregressive error covariance structure was specified for all models. Model fit was compared using the deviance statistic, the Akaike information criterion, and the Schwarz Bayesian criterion. Additionally, analysis of covariance (ANCOVA) models examined weight change between baseline and 4 months after adjusting for baseline weight, sex, and age. We handled missing for the ANCOVA analyses in two ways: (1) a completer analysis included participants with complete baseline and 4-month data only, and (2) an analysis using imputation (last observation carried forward [LOCF]) for participants missing 4-month data. All analyses were conducted using SPSS statistical software, version 12.0 (SPSS Inc, Chicago, IL, USA). All reported *P* values are for two-sided tests, with effects considered statistically significant at *P* < .05.

## Results

A total of 65 participants (mean age 44.9 years, mean weight 89 kg, mean BMI 33.2 kg/m^2^) completed baseline measures and were included in the analyses; 80% were women, 75% were white, and 17% were African American (Table 2). There were no differences in sample characteristics between the intervention and comparison groups, except for mean age—the intervention group was 5 years older (*P* = .007).

**Table 2:** Sample participant characteristics

Characteristic	Comparison (n = 32)	Intervention (n = 33)	Total (n = 65)	*P*
BMI, kg/m^2^ (mean ± SD)	33.5 ± 4.5	32.8 ± 4.3	33.2 ± 4.4	.53
Sex female (%)	27 (84)	25 (76)	52 (80)	.39
Age, years (mean ± SD)	42.4 ± 7.5	47.4 ± 7.1	44.9 ± 7.7	.007
Hispanic (%)	10 (31)	6 (18)	16 (24.6)	.22
Race				.87
White (%)	24 (75)	25 (76)	49 (75)	
African American (%)	4 (13)	7 (21)	11 (17)	
Asian/Pacific Islander (%)	1 (3)	1 (3)	2 (3)	
Prefer not to state (%)	3 (9)	0 (0)	3 (5)	

Sample means for weight status over the course of the study are shown in Table 3. There were no significant differences in weight at baseline between the intervention and comparison groups. At baseline, the comparison group averaged 88 kg, and they did not lose weight between baseline and months 2 and 4. The intervention group averaged 90 kg at baseline and by months 2 and 4, weighed 86 kg and 85 kg, respectively.

**Table 3:** Unadjusted mean weights by groups

	Comparison			Intervention		
	n	Mean Weight (kg)	SD	n	Mean Weight (kg)	SD
Baseline	32	88.02	13.10	33	89.79	17.17
2 months	24	88.03	12.69	28	85.65	16.26
4 months	26	87.85	14.11	26	85.17	18.16

Table 4 shows the results of a mixed-model repeated-measures analysis that assumes any missing data were missing at random. No between-group difference was observed for the adjusted initial weight status. Differences for sex and age were found on adjusted initial weight status; males were 15 kg heavier than females, and age (mean centered) was negatively associated (−0.54 kg per year) with weight. At the end of 4 months, the intervention group lost more weight than the comparison (1.97 kg more; *P* = .02), after adjusting for time, sex, and mean age. The comparison participants’ adjusted weight loss was 0.91 kg. Intervention participants’ adjusted average weight loss was 2.88 kg. This equates to 1.01% and 3.16% weight loss in the comparison and intervention groups, respectively.

**Table 4:** Mixed-effects repeated-measures model parameter estimates for weight change

Primary Outcome Model^a^	Parameter Estimate	SE	*P*	95% CI	
Intercept	82.42	2.17	< .001	78.10	86.75
Group	1.09	3.04	.72	−4.98	7.17
Sex	15.12	3.48	< .001	8.17	22.06
Age	−0.53	0.20	.01	−0.92	−0.13
Time	−0.45	0.30	.13	−1.04	−0.14
Group × Time	−0.98	0.41	.02	−1.80	−0.17

^a^Group is coded as 0 for comparison and 1 for intervention; sex, 0 for female and 1 for male; time, 0 for baseline, 1 for 2 months, 2 for 4 months; age was mean centered.

Table 5 shows adjusted mean weight change for participants with complete data only and for participants where LOCF imputation substituted for missing data at 4 months. For both missing-data methods, the ANCOVA models revealed significantly greater weight loss for the intervention compared to the comparison group after adjusting for baseline weight, sex, and age. When examining participants with complete data, the intervention group lost 1.99 kg more than the comparison group (*P* = .04). The intervention group lost 1.70 kg more than the comparison group when missing values were imputed (*P* = .03).

**Table 5:** Adjusted weight change (kg) at 4 months using two missing-data methods, by group^a^

	Comparison					Intervention					*P*	Eta^2^
	n	Mean	SE	95% CI		n	Mean	SE	95% CI			
Completers only	26	−0.47	0.64	−1.76	0.82	26	−2.46	0.64	−3.75	−1.18	.04	.09
LOCF imputation	32	−0.40	0.51	−1.43	0.63	33	−2.10	0.51	−3.11	−1.09	.03	.08

^a^ANCOVA models adjusted for baseline weight, sex, and age (mean centered). Completers includes participants with non-missing data at both baseline and 4 months only; LOCF, carrying the last observation forward to impute missing 4-month weight.

Adherence was calculated as the percentage of messages requesting a reply that prompted an actual response. During the first week, participants responded to all of the messages that requested a reply. By week 16, participants were responding to approximately two out of three messages. Overall, satisfaction with the intervention was extremely high, with 22 out of 24 (92%) participants stating that they would recommend the intervention to friends and family. Responses to an open-ended question about what users most liked included the following: “The messages served as an excellent reminder to watch what I ate.”; “I found texting my weight every week was extremely helpful.”; “Keeps me focused.”; and “I miss my 6AM message!”

## Discussion

In this pilot study, we found that an intervention based primarily upon the use of tailored SMS messages was effective in promoting weight loss over 4 months among a group of overweight and obese adults. To our knowledge, this is the first randomized controlled trial to examine the use of daily SMS messages for this purpose. While the weight loss for the intervention group was modest (2.88 kg; 3.16%), the loss was robust when examined by different analyses and methods to handle missing data, and it may be meaningful, particularly at a population level. In the Diabetes Prevention Program, every 1 kg of weight loss was associated with a 13% reduction in the risk of incident diabetes [22]. These results compare favorably to an evaluation of eDiets.com, an Internet-based commercial weight loss program that was shown to produce a 0.9% loss of initial weight at 16 weeks [23]. They also compare favorably to the results found with the Internet-only treatment arm of a study comparing it to Internet plus behavioral e-counseling, where those randomized to the Internet-only arm lost, at 12 months, 2.2% of initial body weight, compared to 4.8% for the e-counseling group [24]. Finally, a study comparing mail, phone, and usual care for weight loss among overweight adults in a managed care setting found mean weight losses of 1.93 kg, 2.38 kg, and 1.47 kg at 6 months, respectively, results generally comparable to the findings in the current study [25].

While the intervention has several components (daily text messages, monthly paper materials, and brief phone calls), the cost of deployment was relatively low because the tailoring process is automated, both at program onset and with continued use. Thus, additional users can be added to such a system at a low cost per user.

There are several limitations to the study. Generalizability is limited because of the small sample size and relatively narrow age range of users. The 4-month period of study is short and insufficient to determine if a more clinically important threshold of 5% or 10% weight loss could be achieved. Because the intervention included other components to supplement the text messages, such as monthly print materials and brief monthly phone calls, the specific effects of the daily SMS system cannot be teased out and may limit population reach. However, our experience with brief phone calls and print materials suggests that these components are insufficient to produce the magnitude of weight change observed. Users were already familiar with using text messages and thus may have been of higher literacy and socioeconomic status than many who stand to benefit from weight loss programs. Inferences about whether participants habituate to text messages or whether the 3% weight change would persist over time also cannot be made from this study. On the other hand, the improvements in weight, combined with the favorable impression that the application made with almost all users, suggest the need to continue to explore the value of using daily text message–based interventions for weight control and weight-related behaviors.

There is promise for many health-related uses of mobile phones [26,27], and text messaging stands out since it is one of the only forms of communication that is usable on essentially all types of handsets and with all commercial phone companies. In addition, the popularity of this feature is growing among mobile users [28]. Thus, there are few barriers to the deployment of interventions using this relatively simple mode of communication to reach individuals with health behavior prompts and coaching.

## Abbreviations

BMI: body mass index
MMS: Multimedia Message Service
SMS: Short Message Service
